# Highly-Sensitive Surface-Enhanced Raman Spectroscopy (SERS)-based Chemical Sensor using 3D Graphene Foam Decorated with Silver Nanoparticles as SERS substrate

**DOI:** 10.1038/srep23733

**Published:** 2016-03-29

**Authors:** Chavis Srichan, Mongkol Ekpanyapong, Mati Horprathum, Pitak Eiamchai, Noppadon Nuntawong, Ditsayut Phokharatkul, Pobporn Danvirutai, Erik Bohez, Anurat Wisitsoraat, Adisorn Tuantranont

**Affiliations:** 1 Microelectronics and Embedded Systems Department, Asian Institute of Technology, Pathumtani, 12120, Thailand; 2 Department of Computer Engineering, Khon Kaen University, Khon Kaen, 40002, Thailand; 3 National Electronics and Computer Technology Center (NECTEC), 12120, Thailand

## Abstract

In this work, a novel platform for surface-enhanced Raman spectroscopy (SERS)-based chemical sensors utilizing three-dimensional microporous graphene foam (GF) decorated with silver nanoparticles (AgNPs) is developed and applied for methylene blue (MB) detection. The results demonstrate that silver nanoparticles significantly enhance cascaded amplification of SERS effect on multilayer graphene foam (GF). The enhancement factor of AgNPs/GF sensor is found to be four orders of magnitude larger than that of AgNPs/Si substrate. In addition, the sensitivity of the sensor could be tuned by controlling the size of silver nanoparticles. The highest SERS enhancement factor of ∼5 × 10^4^ is achieved at the optimal nanoparticle size of 50 nm. Moreover, the sensor is capable of detecting MB over broad concentration ranges from 1 nM to 100 μM. Therefore, AgNPs/GF is a highly promising SERS substrate for detection of chemical substances with ultra-low concentrations.

The rise of graphene has opened up many promising applications in various fields due to intriguing relativistic properties of graphene such as massless fermion dispersion, high electron mobility, high conductivity, etc. Recently, there are numerous articles claiming that SERS on graphene could be greatly enhanced in comparison with other substrates. Wang *et al.*^1^ fabricated gold nanoparticles with pyramidal shape onto graphene surface and found that SERS signal of graphene was unusually large^1^. There are few main plausible mechanisms responsible for SERS enhancement on graphene. Firstly, charge transfer from the graphene to nanoparticle could lead to enhanced electric field via localized surface Plasmon (LSP). The second mechanism is based on LSP confinement on graphene. The LSP confinement will result in focusing of electromagnetic wave into smaller area and shorter wavelength (*λ*), which will further enhance the intensity of reflected electromagnetic field and hence SERS signal. The LSP wavelength compression ratio (*λ*/*λ*_*sp*_ where*λ*_*sp*_ = LSP wavelength) due to 2D confinement of LSP on graphene surface could be in the range of 40 ~ 70^2^.

Graphene is also a promising material for chemical sensing due to its excellent physical, chemical and electrical properties including large effective surface area, high electron transfer capability and good chemical stability. For example, electrochemical sensors based on microporous three-dimensional (3D) graphene foam (GF) has demonstrated excellent chemical sensing performances with high sensitivity, low detection limit and good stability^3^,^4^. In addition, GF-based gas sensor is capable of NO_2_ detection at very low concentration^5^. Moreover, LSP resonance (LSPR) from hybrid Ag/GF has been used to catalyze various chemical reactions^6^,^7^,^8^. Since graphene can provide simultaneous sensing enhancements via electromagnetic (EM) and chemical mechanisms, it should be a great platform for SERS-based chemical sensors that can detect trace amount of biochemical analytes or explosives. It has been reported that EM enhancement of graphene is considerably larger than the chemical one^6^. Thus, EM enhancement of graphene and graphene foam with deposited noble metals will be particularly addressed in this study.

According to Kneipp^9^, EM enhancement due to silver nanoparticles on a planar metallic surface can be expressed as^9^


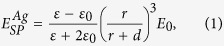


where *ε*_0_ and *ε* are vacuum and metallic-nanomaterial dielectric constants, respectively, *d* is the distance from analyte to silver nanoparticle surface, r is the radius of the metallic nanoparticle, 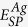
and *E*_0_ are outgoing (enhanced) and incoming electric field intensity, respectively.

The EM enhancement will be analyzed using the LSP model on graphene surface where plasmonically metallic sphere is placed onto the graphene surface as depicted in Fig. 1. Supposing that the oscillating dipole induced on graphene is *P*_*G*_, the electric field on graphene (*E*_*G*_) will be given by


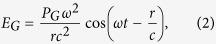


where *ω* = induced dipole frequency and c = light velocity. Assuming that the graphene response latency is negligible, 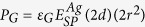 where *ε*_*G*_ = the dielectric constant of graphene. Putting *P*_*G*_ into (2), it can be shown that


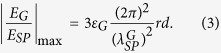


where 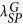 is given by


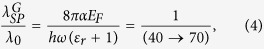


where*λ*_0_ = the wavelength of incident light, *ε*_*r*_ = the dielectric constant of medium, α = fine constant and *E*_*F*_ = Fermi energy of graphene. The enhancement factor (EF) depends strongly on the dimensionless ratio, 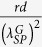. Putting all constants into (3) yields


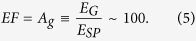


Therefore, the enhancement factor of ∼100 could be achieved on flat graphene.

There are two most common types of chemical sensors: electronic and optical. Optical chemical sensors have recently earned much attention due to short detection time, high selectivity, wide dynamic range and non-contact measurement capability. Among various optical sensing platforms, SERS is becoming increasingly popular due to its extremely-high sensitivity with the detection limit as low as a single molecule and high specificity at ultra-low analyte concentrations. Recently, many chemical sensors using a flat graphene sheet as a SERS substrate have been reported^10^.

Nevertheless, the performances of flat graphene platform should be further improved by employing 3D graphene structure^11^. 3D structure should be useful for SERS-based chemical sensing due to high specific surface area and enhanced optical scattering. The scattered light from a large number of silver nanoparticles situating on multi-layer graphene sheets in graphene foam may be cumulatively integrated, resulting in higher Raman enhancement factor. In this work, graphene foam fabricated by chemical vapor deposition (CVD) using Ni Foam as the catalytic template followed by Ni wet etching using HCl is decorated with silver nanoparticles by direct-current (DC) sputtering and applied as SERS substrate for methylene blue (MB: C_16_H_18_N_3_SCl) detection. In addition, SERS performances are optimized by varying the Ag nanoparticle size via the control of sputtering time.

## Results and Discussion

**Figure 2:**
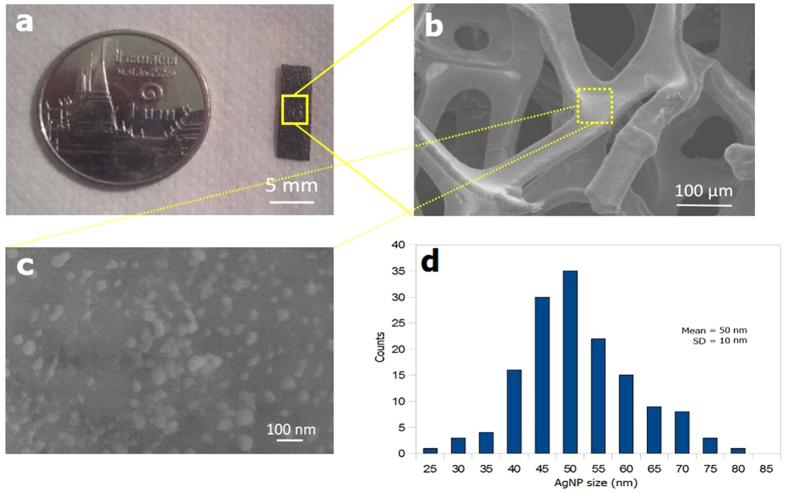
(**a**) Photograph of graphene foam grown from Ni foam scaffold, FESEM images of (**b**) graphene foam at low magnification and (**c**) 30 s-Ag-sputtered graphene foam at high magnification. (**d**) Size distribution of AgNPs on GF.

Figure 2(a) demonstrates a typical CVD-grown graphene foam fabricated from Ni foam scaffold. From the photograph, it is a small rectangular strip (5 × 15 mm^2^) that appears like a thin black sponge. A portion of graphene foam examined by field-emission scanning electron microscopy (FE-SEM) is illustrated in Fig. 2(b). It shows interconnected lamellae with open pores having diameters in the range of 50–300 μm. Figure 2(c) displays the detailed surface morphology of graphene foam after silver sputtering for 30 s. It is seen that there are approximately round nanoparticles scattered on graphene surface. In addition, there are considerable variations of particle size and distribution. The particle size and size distribution are quantitatively evaluated from SEM images and plotted into a histogram as shown in Fig. 2(d). It is seen that the majority of particles have size in a narrow range of 40–55 nm. The mean and standard deviation of particle size are estimated to be ∼50 nm and ∼10 nm, respectively. Thus, the size of sputtered Ag nanoparticles is fairly uniform but the spatial distribution is quite random. The variation may practically acceptable in some applications. However, it could be further improved by optimizing Ag sputtering process parameters to widen the applications of this SERS substrate.

**Figure 3:**
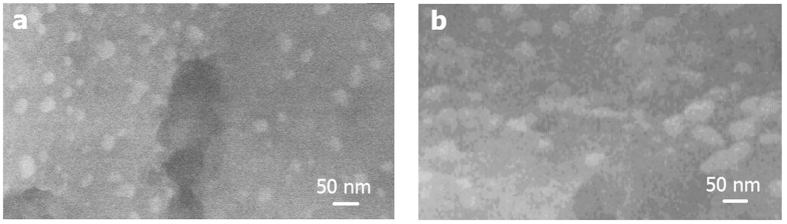
FE-SEM images of AgNPs/GF substrates deposited with Ag sputtering times of (**a**) 20 s and (**b**) 30 s.

**Figure 4:**
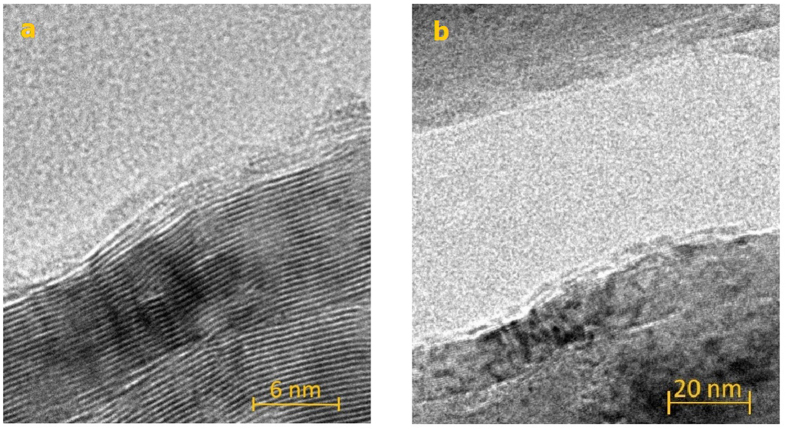
(**a**) HR-TEM and (**b**) BF-TEM images of graphene sheets from graphene foam.

Figure 3 shows the comparison of Ag nanoparticles on graphene foam deposited with two different sputtering times of 20 and 30 s. It is seen that the average particle size increases but the average particle distance decreases with increasing sputtering time due to particle aggregation. Figure 4a,b illustrate typical high-resolution (HR) and bright-field (BF) transmission electron microscopic (TEM) images of graphene foam. It can be seen that CVD graphene foam exhibits smooth edges and comprises 5–10 graphitic fringes on an edge, confirming multi-layer graphene structures.

**Figure 5:**
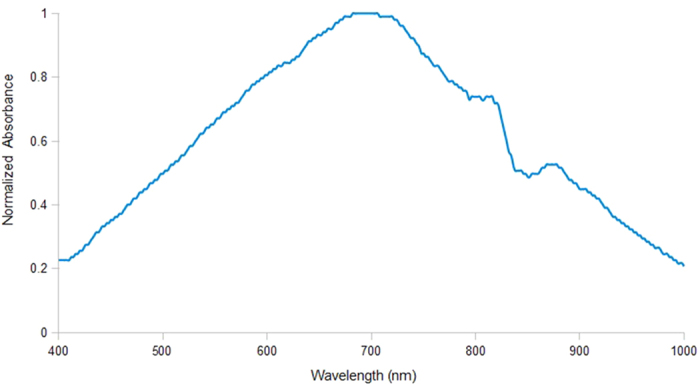
UV-Vis absorption spectrum of 30 s-AgNPs/GF showing a broad LSPR peak centered at ∼700 nm.

To obtain the most effective surface enhanced Raman scattering, the SERS substrate should be excited at its LSPR peak. The typical LSPR peak of AgNPs-GF measured by UV-Vis absorption spectroscopy is demonstrated in Fig. 5. It is seen that the broad LSPR peak is centered at ∼700 nm, which is considerably different from some other reported LSPR peak of AgNPs at ∼450 nm^9^. The difference may be attributed to strong plasmonic coupling between AgNPs and Graphene in this hybrid system. Thus, the excitation wavelength of 785 nm is selected since it is the nearest excitation wavelength of Raman spectrometer available in our laboratory. The relative absorbance is still as high as 0.7 at this wavelength and SERS effect should still be sufficiently effective.

**Figure 6:**
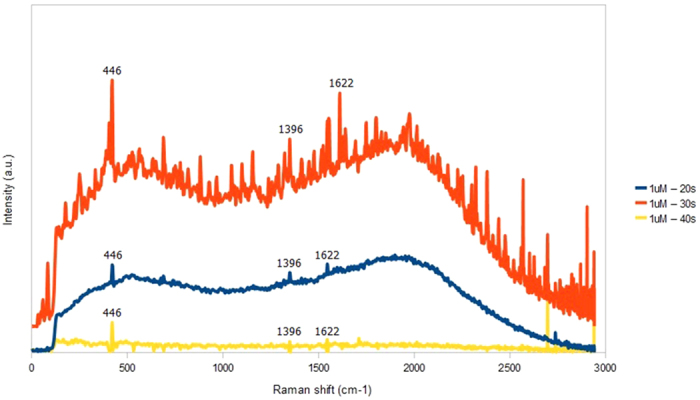
Raman spectra of 1 μM MB on AgNPs/GF substrates prepared with various sputtering times including 20 s, 30 s and 40 s.

Figure 6 shows Raman spectra of 1 μM MB on AgNP/GF fabricated with varying sputtering time from 20 to 40 s. It is seen that the amplitudes of main MB characteristic peaks at 446, 1396 and 1622 cm^−1^ are enhanced substantially as the sputtering time increases from 20 to 30 seconds. In contrast, MB peaks, background signal and noise peaks are greatly diminished when the sputtering time increases further to 40 s. Thus, the optimal sputtering time of AgNPs on GF for the highest MB signal is 30 s. However, the magnitudes of background signal and noise peaks from contaminations also increase considerably compared with the signal peaks. The impurity peaks are also amplified due possibly to the multiple-cascaded amplification process in graphene foam structure and very broad LSPR peak of AgNPs/GF hybrid. Thus, there will be a trade-off between sensitivity and specificity of the SERS sensor based on the AgNPs/GF structure. A practical solution to this problem is to apply a suitable noise filtering system, which can effectively eliminate the majority of noise peaks.

**Figure 7:**
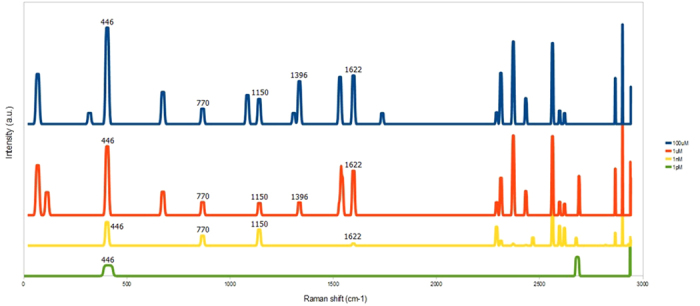
Filtered SERS intensity of MB with different concentrations (100 μM, 1 μM, 1 nM, and 1 pM) on a 30 s-AgNPs/GF substrate.

Silver-decorated graphene foam with the average particle size of 50 nm (30 s sputtering time) was used as SERS substrate to detect MB with varying concentration from 1 pM to 100 μM as demonstrated by SERS spectra in Fig. 7. It should be noted that these SERS spectra were processed by low-pass filtering to eliminate background slope and peak-thresholding to remove several noise peaks. It is seen that five main MB characteristic peaks of at 446, 770, 1150, 1396 and 1622 cm^−1^ can be clearly observed in the SERS spectra for 100 μM and 1 μM MB concentrations while SERS spectra for 1 nM and 1 pM MB concentrations shows only four (446, 770, 1150 and 1622 cm^−1^) and one (446 cm^−1^) MB Raman peaks, respectively. In addition, SERS intensity at a given MB peak increases monotonically as the MB concentration increases from 1 pM to 100 μM. The detection limit for MB is taken to be 1 nm as it is the lowest concentration that four MB peaks can still be observed. The results demonstrate the possibility of using GF platform for detection of trace-level molecules or low concentration of biochemical analytes.

**Figure 8:**
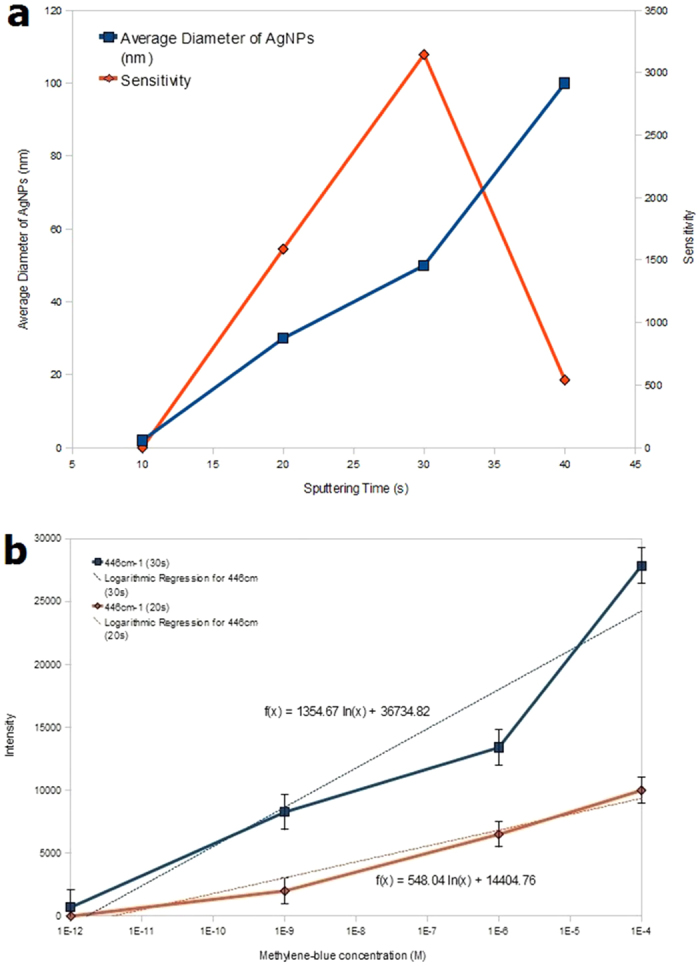
(**a**) Average particle diameter of AgNPs (blue) and sensitivity (red) versus Ag sputtering time and (**b**) Raman intensity at 446 cm^−1^ versus MB concentration plots for AgNPs/GF substrates deposited with Ag sputtering times of 20 s and 30 s.

**Figure 9:**
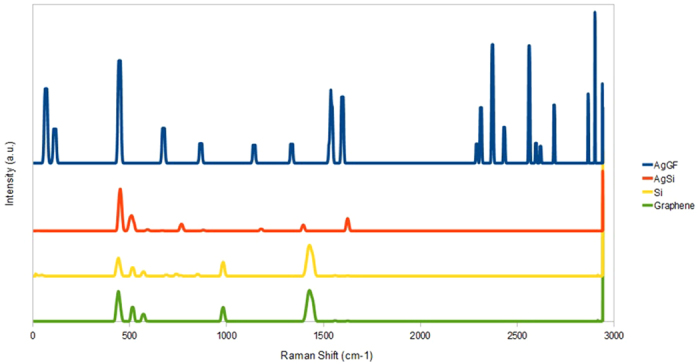
Comparison of Filtered SERS intensity of 1 μM MB on different SERS substrates including AgNPs/GF, AgNPs/Si, Si and graphene.

Figure 8(a) shows the effects of Ag sputtering time on the average particle size and MB sensitivity. The sensitivity is calculated from the fitted logarithmic slope of intensity versus concentration as illustrated in Fig. 8(b). The logarithmic slope is used because the SERS intensity is approximately proportional to MB concentration in logarithmic scale. From Fig. 8(a), it is evident that the sensitivity initially increases as the sputtering time and particle size increase from 0 to 30 s and 0 to 50 nm, respectively. However, the sensitivity decreases substantially when the sputtering time and particle size increases further to 40 s and 100 nm, respectively. Thus, the optimal average particle size that yields the highest sensitivity is 50 nm. The SERS spectra of 1 μM MB on AgNPs/GF substrate is compared with those of AgNPs/Si, Si and graphene substrates as displayed in Fig. 9. It is seen that the AgNPs/GF substrate exhibits higher MB peak amplitudes than all other substrates. In addition, graphene substrates with and without AgNPs always give higher Raman signals than those of corresponding silicon substrates. The EF ratio of GF substrate can be calculated from the ratio of SERS intensities of MB on AgNPs/GF over AgNPs/Si substrates, respectively. From the experimental data, the EF ratio of GF is estimated to be ∼5×10^4^.

From overall results, graphene foam decorated with silver nanoparticles has shown high SERS enhancement factor and excellent MB detection sensitivity. In addition, the Ag sputtering time of 30 s, which produces the average Ag particle size of 50 nm, yields optimal sensitivity for MB detection with a low detection limit of 1 nM. With a short sputtering time (10 s), there is very low density of small Ag nanoparticles. The large distance between small AgNPs will exhibit low local field enhancement and hence low SERS sensitivity. The increase of sputtering time from 10 to 30 s will result in the larger particle size and higher particle density, leading to enhancement of local electric field and LSP. However, AgNPs will become too dense and approaches a thin-film structure as the sputtering time becomes longer than 30 s, resulting in lower surface enhancement capability. In addition, the large particle size will cause non-radiative multimode excitation of LSP^12^, leading to lower surface local field enhancement. Moreover, the AgNPs/GF substrate provides significantly higher SERS enhancement compared with AgNPs/Si, Si and graphene substrates due to geometric and conductivity effects. In order to quantitatively explain the SERS enhancement effect of AgNPs/GF, we propose two distinct mechanisms, namely multiple cascaded amplification and collective excitation.

**Figure 10:**
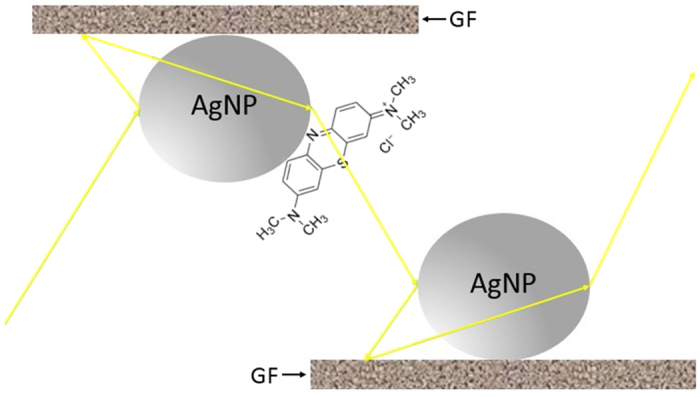
Multiple cascaded amplification scheme for a single molecule on AgNPs/graphene.

**Figure 11:**
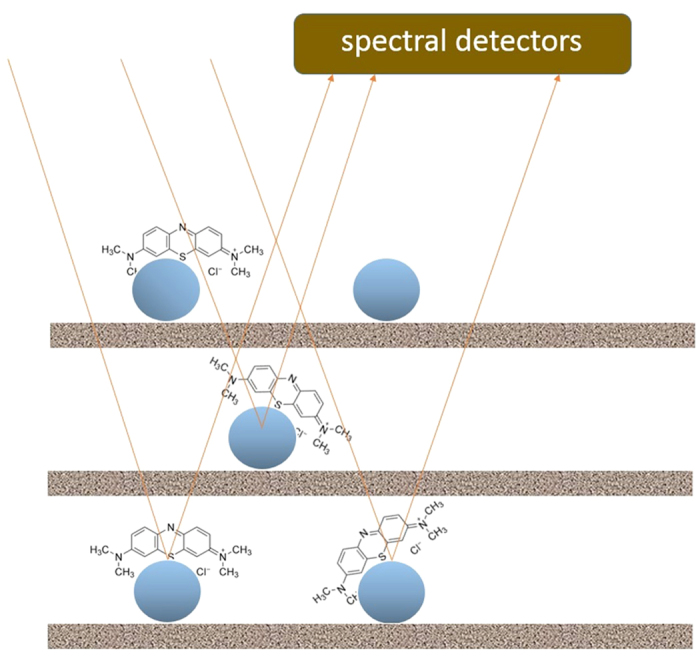
Collective excitation model for many molecules on multilayer of AgNPs/graphene.

In the multiple cascaded amplification process as schematically depicted in Fig. 10, the photon incident on an AgNP will be first amplified by LSP mode focused on the surface of graphene and then further intensified by the charge transfer and LSP 2D confinement on graphene sheet as described earlier. The scattered photon derived from above processes will be further amplified and scattered through another interface of AgNP/Graphene until it finally exits to a photodetector. This mechanism can be used to explain high SERS intensity resulted from a single molecule situated at an AgNP on graphene. The second mechanism is collective excitation of many molecules at multiple sites on AgNPs/graphene layer as schematically described in Fig. 11. This mechanism will be used to account for integration of multiple photons scattered from many analyte molecules located on AgNPs/graphene layers.

According to multiple cascaded amplification scheme, the electromagnetic enhancement factor (EF) can be expressed as


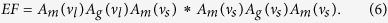


Assuming that


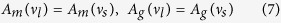


Thus,


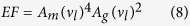


where *A*_*m*_(*ν*_*l*_)^2^ = SERS enhancement factor of silver, typically 10^4^–10^5^, *A*_*m*_(*ν*) = SERS electromagnetic gain of a noble metal (m), *ν* = light frequency, *ν*_*l*_ and *ν*_*s*_ are incident and scattering light frequencies, respectively, and *A*_*g*_(*ν*)^2^ = SERS enhancement factor of graphene^13^, which is typically equal to 10^4^.

In the absence of graphene, EF is defined as EF_0_, which is given by


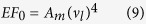


Dividing equations (8) and (9) yields


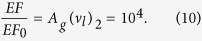


Therefore, the theoretical ratio of enhancement factor for AgNPs substrates with and without GF is 10^4^ which is in the same order of magnitude as the estimated value from the experiment of 5 × 10^4^. However, the other amplification mechanism, the collective excitation model, will also be considered since the first scheme still exhibits considerable discrepancy.

The collective excitation model as depicted in Fig. 11 can be mathematically described as followed.

Define


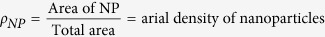


and


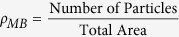


Thus, the amplification gains of graphene (*I*^*G*^) and graphene foam (*I*^*GF*^) can be computed as


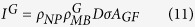


and


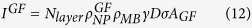


where *γ* is the porosity of GF, *D* is the laser beam spot area, *σ* is the scattering cross section and *A*_*GF*_ is the electromagnetic gain by SERS using GF. Therefore,


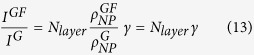


For the AgNPs/GF substrate in this work, GF thickness is 1 mm and inter-layer distance between graphene layers is roughly 0.1 mm. Therefore, the average number of graphene layers (*N*_*layer*_) could be estimated as 1 mm/0.1 mm ~ 10 layers. Thus, the enhancement factor of AgNps/GF substrate based on collective excitation scheme could be ~10^2^ or 100, which is much lower than the enhancement factor from the experimental data of ~5 × 10^4^. Therefore, the multiple cascaded amplification process, which yields a high enhancement factor of 10^4^, is the more likely mechanism for the explanation of large SERS enhancement of AgNPs/GF substrate.

In conclusion, AgNPs/GF has been developed as a novel SERS substrate and applied for detection of MB. The effect of Ag nanoparticle size has been studied by varying Ag sputtering time. The results show that the sputtering time of 30 s produces the average Ag particle size of 50 nm and yields optimal sensitivity for MB detection with a low detection limit of 1 nM. In addition, AgNPs/GF exhibits much high SERS enhancement factor compared with AgNPs/Si, Si and graphene substrates. Cascaded amplification from multiple nanoparticle scattering sites is shown to be the most probable mechanism responsible for the observed enhancement of SERS effect on AgNPs/GF substrate. Therefore, AgNPs/GF is a new promising platform for ultra-sensitive SERS-based sensors that can detect trace-amount of Raman-active molecules such as explosives and other biochemical species.

## Methods

### Material preparation and characterization

Graphene was grown on commercial Ni foams, which were simultaneously utilized as catalyst and scaffold, by chemical vapor deposition (CVD). Acetylene (C_2_H_2_) and hydrogen (H_2_) was used as the carbon precursor and reducing gas carrier for graphene synthesis. C_2_H_2_/ H_2_ (3/24) mixture were flowed through Ni foam located at the center of a horizontal tube furnace under a vacuum pressure of 0.2 Torr at 700 °C for 3 minutes and then cool down rapidly at a rate of more than 10 °C per minutes under hydrogen flow at a pressure of 1 Torr. Carbon atoms from C_2_H_2_ were dissolved on Ni surface and then segregated as graphene layer on Ni scaffold during cooling process. The resulting grahene coated Ni foam was etched in 3 M HCl solution at 60 °C for 30 minutes in order to remove the Ni scaffold. The GF was then decorated with silver nanoparticles (AgNPs) using DC magnetron sputtering with varying sputtering time from 10 to 40 s. The sputtering was conducted in argon at a vacuum pressure of 3 × 10^−3^ mbar with a DC current of 0.2 A.

The structure of graphene sheets from graphene foam was verified by transmission electron microscopy (TEM: JEOL, JEM-2010). The surface morphology of Ag-decorated graphene foam was examined using field emission scanning electron microscopy (FESEM: Hitachi, SU-8030). The optical absorption spectrum of AgNPs-GF was measured by UV-Vis absorption spectrometer (Shimadzu, UV3101PC).

### SERS measurement

SERS measurement was conducted using a portable Raman spectrometer (Enwave Optronics Field-type EZRaman-M) with the laser wavelength of 785 nm (red) and reflection detection at zero degree. Before MB detection, Raman spectrum of SERS substrate was measured in order to determine the baseline signal. Afterwards, MB solution was dropped onto a SERS substrate and then exposed to the laser in order to take Raman spectrum. Firstly, SERS measurement was carried out at a fixed MB concentration of 1 μm on AgNPs/GF substrates prepared with different Ag sputtering times (10–40 s) in order to determine the optimal Ag deposition time. Next, the SERS measurements were repeatedly performed on each substrate with varying MB concentration from 1 pM to 100 μM to obtain calibration curve and sensitivity. Lastly, SERS experiments were done on different SERS substrates including AgNPs/GF, AgNPs/Si, Si, and graphene at a fixed MB concentration of 1 μM in order to compare the performances of AgNPs/GF substrates and determine the enhancement factor derived from GF structure. It should be noted that the graphene substrate used for the comparison was a planar CVD graphene layer grown on a copper foil.

## Additional Information

**How to cite this article**: Srichan, C. *et al.* Highly-Sensitive Surface-Enhanced Raman Spectroscopy (SERS)-based Chemical Sensor using 3D Graphene Foam Decorated with Silver Nanoparticles as SERS substrate. *Sci. Rep.* **6**, 23733; doi: 10.1038/srep23733 (2016).

## Figures and Tables

**Figure 1:**
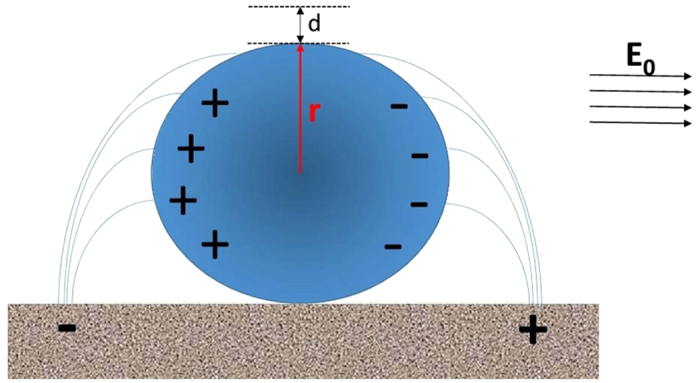
Localized Surface Plasmon model on graphene surface.
